# Cases of antiretroviral-associated gynaecomastia reported to the National HIV & Tuberculosis Health Care Worker Hotline in South Africa

**DOI:** 10.1186/s12981-016-0121-z

**Published:** 2016-11-16

**Authors:** Christine Njuguna, Annoesjka Swart, Marc Blockman, Gary Maartens, Briony Chisholm, Annemie Stewart, Anri Uys, Karen Cohen

**Affiliations:** Division of Clinical Pharmacology, Department of Medicine, University of Cape Town, Cape Town, South Africa

**Keywords:** Gynaecomastia, Antiretroviral therapy, Efavirenz, South Africa

## Abstract

**Background:**

Gynaecomastia is associated with exposure to antiretroviral therapy (ART), in particular efavirenz. There is limited data on clinical characteristics of patients with ART-associated gynaecomastia in resource-limited settings and little guidance on the optimal management of this adverse drug reaction (ADR). We describe the clinical characteristics, management and outcomes of gynaecomastia cases reported to the National HIV & Tuberculosis Health Care Worker Hotline in South Africa.

**Methods:**

We identified all gynaecomastia cases in adolescent boys and men on ART reported to the hotline between June 2013 and July 2014. We collected follow up data telephonically at monthly intervals to document clinical management and outcomes.

**Results:**

We received 51 reports of gynaecomastia between June 2013 and July 2014; 11% of the 475 patient-specific ADR queries to the hotline. All patients were on efavirenz-based ART. Mean age was 34 years (standard deviation 12) and seven were adolescents. The median onset of gynaecomastia was 15 months after efavirenz initiation (interquartile range 6–42). Gynaecomastia was bilateral in 29 patients (57%) and unilateral in 16 (31%). Serum testosterone was quantified in 25 of 35 patients with follow up data, and was low in 2 (8%). Efavirenz was replaced with an alternative antiretroviral in 29/35 patients (83%) and gynaecomastia improved in 20/29 (69%).

**Conclusions:**

Gynaecomastia was a frequently reported ADR in our setting, occurring with prolonged efavirenz exposure. Testosterone was low in the minority of tested cases. Most clinicians elected to switch patients off efavirenz, and gynaecomastia improved in the majority.

## Background

Gynaecomastia, a benign proliferation of glandular breast tissue in males [1] is thought to result from an imbalance between oestrogens and androgens [1]. There are reports of gynaecomastia in HIV infected men before antiretroviral therapy (ART) was available, [2] but most reports are in men on ART. The estimated prevalence ranges from 1.8 to 2.9% [3–5].

Early case reports and case series implicated nucleoside reverse transcriptase inhibitors (NRTIs) and protease inhibitors [5–8]. However, case control studies found that gynaecomastia was associated with exposure to the non-nucleoside reverse transcriptase inhibitor (NNRTI) efavirenz, and the NRTIs stavudine and didanosine [3, 4, 9]. Most reports of ART-associated gynaecomastia come from resource-rich settings, and there are few data from Africa and other resource-limited settings.

We describe the clinical characteristics of HIV infected patients with ART-associated gynaecomastia reported to the National HIV & Tuberculosis (TB) Health Care Worker Hotline in South Africa, clinical investigation and management, clinical outcomes and time to improvement and/or resolution of gynaecomastia.

## Methods

We included all suspected gynaecomastia cases reported to the National HIV & TB Health Care Worker Hotline between June 2013 and July 2014. The hotline is based at the Medicines Information Centre, University of Cape Town, and provides telephonic advice to health care workers on the management of patients with HIV and/or tuberculosis, including adverse drug reactions (ADRs). Details of the service have been described elsewhere [10].

We used a standardized data collection form to collect data on patient characteristics, clinical characteristics of gynaecomastia, serum testosterone results and medication history. We recorded the date of initiation of the current ART regimen and when gynaecomastia was first noted by the patient. We calculated time to onset of gynaecomastia as the time interval from initiation of the current ART regimen to the date when gynaecomastia was first noted by the patient. In four cases where the date when gynaecomastia was first noted was missing, we used the date of the hotline query to calculate time to gynaecomastia onset. We obtained follow-up information telephonically from the health care worker at one month and then monthly or at the next patient visit for at least 6 months. We classified outcomes as resolved, improved (reduction in breast size and/or pain), unchanged, deteriorated or unknown. All descriptive analysis was performed using STATA version 13.

## Results

### Patient characteristics

The hotline received queries from health care workers about suspected gynaecomastia in 51 males between June 2013 and July 2014. This constituted 11% of the 475 patient-specific ADR queries received by the hotline during this period. The mean age of patients with suspected gynaecomastia was 34 years (SD 12) (Table 1). Adolescents comprised 14% of the case series. At the time of reporting gynaecomastia to the hotline, 30/31 (97%) patients with a viral load result recorded were virologically suppressed (Table 1). Gynaecomastia onset was slow, occurring after a median of 15 months, (IQR 6–42) on ART. Gynaecomastia was bilateral in 57% of patients (Table 1).

**Table 1:** Patient characteristics and description of suspected gynaecomastia cases

Patient characteristic	N (%) (n = 51)
Age (years), mean ± SD	34 ± 12
Age category	
Adolescent (10–17 years)	7 (14%)
Adult (≥18 years)	36 (71%)
Unknown	8 (16%)
Baseline CD4 count before ART initiation (mm^3^), mean ± SD	188 ± 95
Current viral load	
Viral load (<50)	26 (51%)
Viral load (51–399)	4 (8%)
Viral load >400	1 (2%)
Viral load unknown	20 (39%)
Characteristics of gynaecomastia	
Site	
Unilateral	16 (31%)
Bilateral	29 (57%)
Unknown	6 (12%)
Breast pain present	10 (20%)
Gynaecomastia onset (months on ART), median [IQR]	15 [6–42]

### Antiretroviral regimens and concomitant drugs

All gynaecomastia cases reported to the hotline were on efavirenz-containing ART. The NRTI backbone at the time of gynaecomastia onset was tenofovir and lamivudine/emtricitabine in 40 (78%) of patients, stavudine and lamivudine in 5 (10%), abacavir and lamivudine in 4 (8%), zidovudine and lamivudine in 1 (2%) of patients and unknown NRTI backbone in 1 (2%) patient.

Sixteen patients (31%) were taking concomitant drugs previously described to cause gynaecomastia in addition to efavirenz: isoniazid in 11 patients, isoniazid and stavudine in one patient, amlodipine and stavudine in one patient and stavudine in three patients.

### Clinical investigations and patient outcomes

We obtained follow-up data on 35 patients (69%) with a median follow-up period of 4 months, (IQR 1–6).

Serum testosterone testing was performed in 25/35 (71%) patients, of which 19 were within the normal range, two cases had low testosterone, two cases had elevated testosterone and in two cases the result was not recorded.

Twenty nine patients (83%) discontinued efavirenz: 27 patients were switched to nevirapine and two patients were switched to lopinavir/ritonavir. Gynaecomastia resolved completely in 7 of these 29 patients, improved in 13 and was unchanged in two, with unknown outcome in 7.

Six patients continued efavirenz. Gynaecomastia improved in one patient and was unchanged in one patient, with unknown outcome in four patients.

**Fig. 1:**
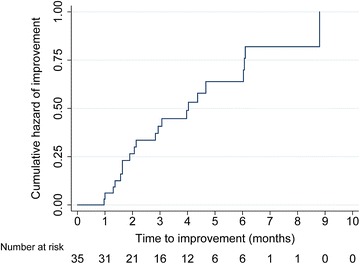
Kaplan Meier curve of time to improvement in patients with follow-up (n = 35)

Median time to first improvement of gynaecomastia was 3 months (IQR 2–4, range1–8) (Fig. 1). Complete resolution occurred after a median of 6 months (IQR 4–6, range 4–9).

## Discussion

We describe a case series of 51 patients with gynaecomastia on efavirenz-based ART in South Africa. Efavirenz is the recommended NNRTI in the standard first-line ART regimen for adults in South Africa [11]. These cases were reported to the National HIV & TB Health Care Worker Hotline and comprised 11% of patient-specific ADR queries over a 13 month period. In the 35 patients with follow-up data, the majority switched from efavirenz to an alternative antiretroviral, and the gynaecomastia improved after efavirenz withdrawal in 69%.

This is to our knowledge the largest case series of ART-associated gynaecomastia cases reported from Sub-Saharan Africa. A small case series from Nigeria reported on six patients with efavirenz-associated gynaecomastia, all of which resolved after withdrawal of efavirenz [12]. Two cases of efavirenz-associated gynaecomastia were reported from Malawi, both of which improved after switching efavirenz to nevirapine [13].

Efavirenz is known to be associated with gynaecomastia [3, 4, 9]. The proposed mechanism from in vitro data is that efavirenz mimics the effects of oestrogen on breast tissue [14]. Two other hypothesized mechanisms have been suggested for ART-associated gynaecomastia: immune restoration may increase breast tissue oestrogen availability, and efavirenz has been shown to increase the area under the curve of ethinyl oestradiol by at least 37%, thereby elevating the oestrogen–androgen ratio [15].

Three cohort studies from Spain and Italy have reported ART-associated gynaecomastia prevalence between 1.8 and 2.9% [3–5], whilst the incidence from another Spanish study of 1400 HIV infected men followed up over 20 months was 2.4 per 100 person years on ART [6]. A study from Nigeria of 2920 HIV infected patients reported an incidence of gynaecomastia of 10.7 per 100 person years over 8 years [16].

Interestingly 7 (14%) of our case series were adolescents. In three of these adolescent patients, gynaecomastia improved after withdrawal of efavirenz, suggesting that efavirenz was a probable contributing cause. However, the physiological changes of puberty may also have contributed to gynaecomastia in the adolescent cases we describe.

In contrast, previous case series do not report any cases of gynaecomastia in adolescents [6, 7, 12, 15, 17–19]. Cross-sectional data from the general population has shown that gynaecomastia occurs in 4% of adolescent boys aged 10–19 years [20]. There is limited data on ART-associated gynaecomastia in African children and adolescents [21, 22].

The clinical presentation of the gynaecomastia in our case series was primarily bilateral after several months on ART, as previously reported in other studies [6, 7, 9, 12]. Previous studies have reported virological suppression in 77–100% of patients at the time of onset of gynaecomastia [6, 8, 12, 15, 19]. In our case series 31 of the 51 patients had a current viral load result at the time of reporting gynaecomastia to the hotline, with viral load <400 copies/mL in 30/31 (97%).

The majority of patients were switched from efavirenz, after which gynaecomastia improved/resolved in the majority. This finding is similar to other case series where efavirenz was discontinued [12, 13, 15], but it is noteworthy that other studies have reported improvement in patients, despite continuation of efavirenz [4, 8, 9]. Time to complete resolution was slow in our case series and was comparable to other studies that have reported a resolution time ranging from two to nine months [4, 6, 8, 12, 15].

Where it was quantified, the free testosterone was normal in the majority of our gynaecomastia cases. In contrast, Biglia et al. reported an association between hypogonadism and gynaecomastia [3]. Typically, the hotline recommends that a free testosterone be done to exclude hypogonadism before switching efavirenz to nevirapine. Free testosterone quantification is not routinely available in many resource-limited settings. The usefulness of testosterone testing in African patients with efavirenz-associated gynaecomastia needs to be explored further.

This case series has limitations. There was limited data on the method of diagnosis of the gynaecomastia cases. Follow-up information could not be obtained on all patients. Information on the exclusion of other causes of gynaecomastia was not always available. Reports were collected and follow-up was done telephonically; hence patient histories, clinical findings and outcomes could not be verified by clinical assessment or by review of patient records. The cases we describe are not a random and unbiased sample of gynaecomastia cases in our setting; they are cases in which a health care worker requested assistance from the hotline advice service.

## Conclusions

In conclusion, efavirenz-associated gynaecomastia is frequently the subject of ADR queries to the HIV & TB Health Care Worker Hotline, suggesting that health care workers in South Africa need guidance on the management of this ADR. In most cases, gynaecomastia cases reported to the hotline occurred after prolonged efavirenz exposure. Efavirenz was withdrawn in the majority of patients, in accordance with advice given by the hotline, and gynaecomastia improved slowly in the majority.

Further studies are needed to determine the incidence and risk factors of ART-associated gynaecomastia in African adolescents and adult men, the role of hypogonadism in gynaecomastia in this setting, and the optimal clinical management of gynaecomastia. Studies answering these questions are essential in order to provide evidence-based guidance to health care workers on the optimal investigation and management of this important treatment-limiting ADR.

## Abbreviations

ADR: adverse drug reaction
ART: antiretroviral therapy
NNRTI: non-nucleoside reverse transcriptase inhibitor
NRTI: nucleoside reverse transcriptase inhibitor
TB: tuberculosis

## References

[1] Barros AC, Sampaio Mde C (2012). Gynecomastia: physiopathology, evaluation and treatment. Sao Paulo Med J.

[2] Couderc LJ, Clauvel JP (1987). HIV-infection-induced gynecomastia. Ann Intern Med.

[3] Biglia A, Blanco JL, Martinez E, Domingo P, Casamitjana R, Sambeat M, Milinkovic A, Garcia M, Laguno M, Leon A (2004). Gynecomastia among HIV-infected patients is associated with hypogonadism: a case-control study. Clin Infect Dis.

[4] Mira JA, Lozano F, Santos J, Ramayo E, Terron A, Palacios R, Leon EM, Marquez M, Macias J, Fernandez-Palacin A (2004). Gynaecomastia in HIV-infected men on highly active antiretroviral therapy: association with efavirenz and didanosine treatment. Antivir Ther.

[5] Manfredi R, Calza L, Chiodo F (2004). Another emerging event occurring during HIV infection treated with any antiretroviral therapy: frequency and role of gynecomastia. Infez Med.

[6] Garcia-Benayas T, Blanco F, Martin-Carbonero L, Valencia E, Barrios A, Gonzalez-Lahoz J, Soriano V (2003). Gynecomastia in HIV-infected patients receiving antiretroviral therapy. AIDS Res Hum Retroviruses.

[7] Peyriere H, Mauboussin JM, Rouanet I, Merle C, Sotto A, Arnaud A, Hillaire-Buys D, Balmes P (1999). Report of gynecomastia in five male patients during antiretroviral therapy for HIV infection. AIDS.

[8] Qazi NA, Morlese JF, King DM, Ahmad RS, Gazzard BG, Nelson MR (2002). Gynaecomastia without lipodystrophy in HIV-1-seropositive patients on efavirenz: an alternative hypothesis. AIDS.

[9] Rahim S, Ortiz O, Maslow M, Holzman R (2004). A case-control study of gynecomastia in HIV-1-infected patients receiving HAART. AIDS Read.

[10] Chisholm BS, Cohen K, Blockman M, Kinkel HF, Kredo TJ, Swart AM (2011). The impact of the National HIV Health Care Worker Hotline on patient care in South Africa. AIDS Res Ther.

[11] National Department of Health. National consolidated guidelines. The prevention of mother-to-child transmission of HIV (PMTCT) and the management of HIV in children, adolescents and adults. 2015. http://www.sahivsoc.org/upload/documents/ART%20Guidelines%2015052015.pdf. Accessed 11 May 2016.

[12] Agbaji OO, Agaba PA, Ekeh PN, Sule HM, Ojoh RO, Audu E, Yiltok SJ, Osho PO, Idoko JA, Kanki P (2011). Efavirenz-induced gynaecomastia in HIV-infected Nigerian men: a report of six cases. J Med Med Sci.

[13] Kwekwesa A, Kandionamaso C, Winata N, Mwinjiwa E, Joshua M, Garone D, Bedell R, van Oosterhout JJ (2015). Breast enlargement in Malawian males on the standard first-line antiretroviral therapy regimen: case reports and review of the literature. Malawi Med J.

[14] Sikora MJ, Rae JM, Johnson MD, Desta Z (2010). Efavirenz directly modulates the oestrogen receptor and induces breast cancer cell growth. HIV Med.

[15] Jover F, Cuadrado JM, Roig P, Rodriguez M, Andreu L, Merino J (2004). Efavirenz-associated gynecomastia: report of five cases and review of the literature. Breast J.

[16] Abah IO, Akanbi M, Abah ME, Finangwai AI, Dady CW, Falang KD, Ebonyi AO, Okopi JA, Agbaji OO, Sagay AS (2015). Incidence and predictors of adverse drug events in an African cohort of HIV-infected adults treated with efavirenz. Germs.

[17] Schinina V, BusiRizzi E, Zaccarelli M, Carvelli C, Bibbolino C (2002). Gynecomastia in male HIV patients MRI and US findings. Clin Imaging.

[18] Evans DL, Pantanowitz L, Dezube BJ, Aboulafia DM (2002). Breast enlargement in 13 men who were seropositive for human immunodeficiency virus. Clin Infect Dis.

[19] Caso JA, Prieto Jde M, Casas E, Sanz J (2001). Gynecomastia without lipodystrophy syndrome in HIV-infected men treated with efavirenz. AIDS.

[20] Kumanov P, Deepinder F, Robeva R, Tomova A, Li J, Agarwal A (2007). Relationship of adolescent gynecomastia with varicocele and somatometric parameters: a cross-sectional study in 6200 healthy boys. J Adolesc Health.

[21] Dzwonek A, Clapson M, Withey S, Bates A, Novelli V (2006). Severe gynecomastia in an African boy with perinatally acquired human immunodeficiency virus infection receiving highly active antiretroviral therapy. Pediatr Infect Dis J.

[22] Tukei VJ, Asiimwe A, Maganda A, Atugonza R, Sebuliba I, Bakeera-Kitaka S, Musoke P, Kalyesubula I, Kekitiinwa A (2012). Safety and tolerability of antiretroviral therapy among HIV-infected children and adolescents in Uganda. J Acquir Immune Defic Syndr.

